# 2-Iminobiotin Superimposed on Hypothermia Protects Human Neuronal Cells from Hypoxia-Induced Cell Damage: An *in Vitro* Study

**DOI:** 10.3389/fphar.2017.00971

**Published:** 2018-01-11

**Authors:** Karina Zitta, Cacha Peeters-Scholte, Lena Sommer, Matthias Gruenewald, Lars Hummitzsch, Kerstin Parczany, Markus Steinfath, Martin Albrecht

**Affiliations:** ^1^Department of Anesthesiology and Intensive Care Medicine, University Hospital Schleswig-Holstein, Kiel, Germany; ^2^Neurophyxia B.V., 's-Hertogenbosch, Netherlands

**Keywords:** hypoxia-ischemia, hypothermia, neuroprotection, asphyxia, 2-iminobiotin, cell damage, apoptosis, *in vitro*

## Abstract

Perinatal asphyxia represents one of the major causes of neonatal morbidity and mortality. Hypothermia is currently the only established treatment for hypoxic-ischemic encephalopathy (HIE), but additional pharmacological strategies are being explored to further reduce the damage after perinatal asphyxia. The aim of this study was to evaluate whether 2-iminobiotin (2-IB) superimposed on hypothermia has the potential to attenuate hypoxia-induced injury of neuronal cells. *In vitro* hypoxia was induced for 7 h in neuronal IMR-32 cell cultures. Afterwards, all cultures were subjected to 25 h of hypothermia (33.5°C), and incubated with vehicle or 2-IB (10, 30, 50, 100, and 300 ng/ml). Cell morphology was evaluated by brightfield microscopy. Cell damage was analyzed by LDH assays. Production of reactive oxygen species (ROS) was measured using fluorometric assays. Western blotting for PARP, Caspase-3, and the phosphorylated forms of akt and erk1/2 was conducted. To evaluate early apoptotic events and signaling, cell protein was isolated 4 h post-hypoxia and human apoptosis proteome profiler arrays were performed. Twenty-five hour after the hypoxic insult, clear morphological signs of cell damage were visible and significant LDH release as well as ROS production were observed even under hypothermic conditions. Post-hypoxic application of 2-IB (10 and 30 ng/ml) reduced the hypoxia-induced LDH release but not ROS production. Phosphorylation of erk1/2 was significantly increased after hypoxia, while phosphorylation of akt, protein expression of Caspase-3 and cleavage of PARP were only slightly increased. Addition of 2-IB did not affect any of the investigated proteins. Apoptosis proteome profiler arrays performed with cellular protein obtained 4 h after hypoxia revealed that post-hypoxic application of 2-IB resulted in a ≥ 25% down regulation of 10/35 apoptosis-related proteins: Bad, Bax, Bcl-2, cleaved Caspase-3, TRAILR1, TRAILR2, PON2, p21, p27, and phospho Rad17. In summary, addition of 2-IB during hypothermia is able to attenuate hypoxia-induced neuronal cell damage *in vitro*. Combination treatment of hypothermia with 2-IB could be a promising strategy to reduce hypoxia-induced neuronal cell damage and should be considered in further animal and clinical studies.

## Introduction

Hypoxic-ischemic encephalopathy (HIE) is a major reason for neonatal death and long-term disabilities. It is the second largest cause of the neonatal global burden of disease and accounts for 2.0% of total disability-adjusted life years (https://vizhub.healthdata.org/gbd-compare). HIE occurs in Western countries in 2-3 cases per 1000 term live born (Gunn and Gunn, 1997). After years of research on cooling, therapeutic hypothermia (33.5°C) became as currently standard of care for newborn infants presenting HIE (Higgins et al., 2011). However, about 45% of infants still continue to have an adverse outcome despite this treatment (Bel, 2016). Therefore, it is a major challenge to develop adjunct therapies on top of hypothermia that will help to reduce the adverse effects of HIE (Martinello et al., 2017).

In the past, several pharmacological therapies were proposed to reduce neonatal brain injury (Robertson et al., 2012; Hassell et al., 2015; Tataranno et al., 2015). Nevertheless, at present no specific alternative therapy exists that effectively reduces neonatal brain injury or ameliorates detrimental neurodevelopmental effects. Therefore, the multimodal approach of combining hypothermia, as established treatment for HIE, together with other neuroprotective pharmacological candidate molecules has gained major interest in the last years (Perrone et al., 2013).

2-iminobiotin (2-IB) is a biotin (also called vitamin H or B7) analog. Structurally 2-IB and L-Arginin are very similar and both contain guanidino and free carboxyl groups (Sup et al., 1994). From *in vitro* studies it was shown that 2-IB represents a selective inhibitor of neuronal and inducible nitric oxide synthase (nNOS and iNOS) (Sup et al., 1994; Peeters-Scholte et al., 2002a,b). Moreover, some authors have proposed antioxidative effects of 2-IB (Peeters-Scholte et al., 2002c; Tataranno et al., 2015) in different models, although no direct intrinsic antioxidative activity of the molecule has been described so far.

We have demonstrated that 2-IB, when administered after hypoxia-ischemia is able to reduce neuronal cell damage in an *in vitro* neuronal cell model of hypoxia-ischemia during normothermia (Zitta et al., 2016). Both, in a rat model of perinatal hypoxia-ischemia as well as in a newborn piglet model, this neuroprotective effect of 2-IB under normothermia was confirmed (van den Tweel et al., 2005; Bjorkman et al., 2013). Furthermore, 2-IB has been administered in two small phase 2 clinical studies under normothermia for safety and pharmacokinetics (www.clinicaltrials.gov: NCT01626924 and www.clinicaltrialsregister.eu: 2015-003063-12).

Since cooling is currently the state-of-the-art treatment for HIE, potential drugs for the treatment of HIE should be tested on top of therapeutic hypothermia. However, drug pharmacokinetics and pharmacodynamics can change dramatically under hypothermic conditions, reducing the drug's efficacy or even leading to adverse events in the respective patients (Sunjic et al., 2015; Anderson et al., 2016). Therefore, prior to its clinical application, the effectiveness of a specific drug on top of hypothermia has to be carefully analyzed using *in vitro* and/or animal models.

Employing our recently described *in vitro* hypoxia model (Zitta et al., 2010a,b; Huang et al., 2013a,b), the current study was designed to (i) evaluate whether 2-IB conveys neuroprotection if superimposed on hypothermia (ii) to estimate which concentrations of 2-IB are most effective in protecting neuronal cells from hypoxia-induced damage and (iii) to gain insight into the possible cellular mechanisms of 2-IB.

## Materials and methods

### Experimental setup

**Figure 1:**
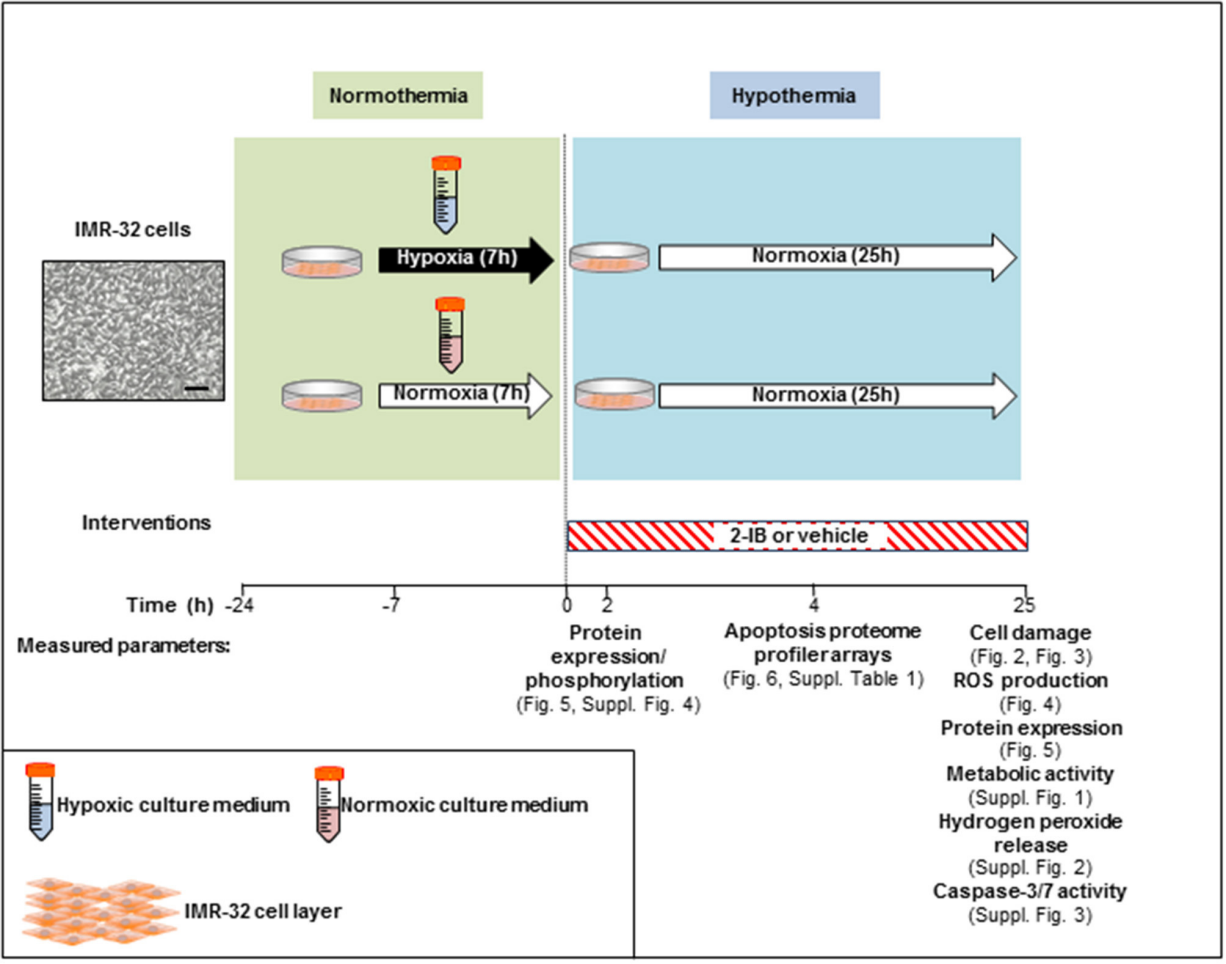
Experimental setup.

*In vitro* hypoxia was induced for 7 h in IMR-32 cell cultures by using our recently described system with minor modifications (Zitta et al., 2010a,b; Huang et al., 2013a,b). Enzyme stock solutions (100x) of catalase and glucose oxidase (both from Sigma-Aldrich, Munich, Germany) were diluted in cell culture medium (DMEM/F12, 1% FCS; final concentration: 120 and 2 U/ml respectively). A rapid decrease of partial pressure of oxygen (pO_2_) to levels below 10 mmHg was achieved by adding the enzymes to glucose containing culture medium. Also a decline in glucose (<1 g/l) and pH (<7.0) was observed, resembling the clinical characteristics of hypoxic-ischemic injury *in vivo* (Huang et al., 2013a). Hypoxic conditions were confirmed with a tissue oxygen pressure monitor (LICOX® CMP Oxygen Catheter; Integra, Plainsboro, USA). After the hypoxic insult, cells were washed twice with PBS (ThermoFisher, Waltham, MA, USA) and cultures were placed into an incubator at 33.5°C (hypothermia) employing culture medium with (i) solvent (citrate buffer 1%) or (ii) 2-IB (2-iminobiotin; IUPAC name: 5-[(3aS,4S,6aR)-2-imino-hexahydro-1H-thieno[3,4-d]imidazolidin-4-yl]pentanoic acid; kindly provided by Neurophyxia, ′s-Hertogenbosch, The Netherlands) at 10, 30, 50, 100, and 300 ng/ml. To determine the optimal “reperfusion” time, a time-interval curve investigating cell damage (LDH release) was performed. Analyses of LDH release, ROS generation, hydrogen peroxide release, metabolic activity, cell signaling, apoptosis-related protein expression/activity and expression analysis of 35 human apoptosis-related proteins were performed at different time points post-hypoxia (Figure 1).

### Lactate dehydrogenase (LDH) cytotoxicity assays and evaluation of confluence of IMR-32 cultures

The release of LDH from cells into the culture medium was quantified by using a colorimetric cytotoxicity detection kit (Roche, Mannheim, Germany) at different time points after the end of hypoxia and normoxia, respectively. Samples were prepared based on the manufacturer's protocol. Briefly, culture media were collected and stored at −20°C. For evaluation of total LDH, cells were lysed with 2% Triton X-100 (Carl Roth, Karlsruhe, Germany) for 15 min at 37°C. LDH activity of the samples was measured in 96-well plates at 490 nm using an ELISA reader (Tecan, Crailsheim, Austria) in combination with the Magellan software v1.1. The increase in LDH release was calculated based on the LDH versus total LDH values in each group. Effects of hypoxia and 2-IB on cell confluence (i.e., area of the culture dish that is not covered by cells) was evaluated using brightfield microscopy in combination with the Image J software (v1.410, NIH) and is shown as percentage of the total culture area.

### Metabolic activity assays

The relative numbers of metabolically active cells were evaluated by using a colorimetric kit (CellTiter96 AQ_ueous_ One Solution Reagent G3580 Promega Madison, WI, USA). Samples were prepared concerning to the manufacturer's protocol. Metabolic active cells generate a colored product whose absorbance was evaluated at 490 nm using an ELISA reader (Tecan, Crailsheim, Austria) in combination with the Magellan software v1.1.

### Measurements of reactive oxygen species

For reactive oxygen species (ROS) measurements, 4 × 10^4^ cells were seeded per well and cultured for 3 days in black walls 96-well plates (Greiner Bio-One, Frickenhausen, Germany) with 100 μl DMEM/F12 supplemented with 10% FCS per well. After 2 days in culture, cells were washed with pre-warmed PBS and cultured in a phenol red-free DMEM/F12 (ThermoFisher, Waltham, MA, USA) with 1% fetal calf serum (PAA). Experiments were performed as described above and intracellular ROS were measured by adding 2′7′-dichlorodihydrofluorescein diacetate (H_2_DCFDA; Sigma-Aldrich), in a final concentration of 10 μM. Fluorescence signals were evaluated with an excitation wavelength of 485 nm and emission wavelength of 535 nm using an ELISA reader (Genios FL; Tecan, Crailsheim, Austria). Fluorescence data were acquired at 0 (T0) and 30 min (T30) time points and results were calculated as the increment of fluorescence (%) over time [(T30-T0)/T0x100].

### Hydrogen peroxide assays

The formation of hydrogen peroxide was measured with a QuantiChrom™ Peroxide Assay Kit (Bio-Assay Systems, Hayward, CA, USA), which utilizes the chromogenic Fe^3+^-xylenol orange reaction, in which a purple complex is formed when Fe^2+^ provided in the reagent is oxidized to Fe^3+^ by hydrogen peroxide present in the sample. Briefly, 100 μl of detection reagent were added to 20 μl of sample and measurements were performed based on the manufacturer's protocol. Samples were evaluated after 30 min in a 96-well plate at 620 nm using an ELISA reader (Tecan, Crailsheim, Austria). Standard curves were created from known concentrations of hydrogen peroxide.

### Western blotting

Protein isolation and extraction was performed using RIPA buffer containing 150 mM sodium chloride, 1% NP-40, 1% sodium deoxycholate, 0.1% sodium dodecyl sulfate (SDS) and 50 mM Tris-HCl (pH 7.6; all from Sigma-Aldrich). Protein concentrations were determined with Roti®-Quant assays (Carl Roth). 30 μg of protein were mixed with 4x Laemmli buffer (8% SDS, 40% glycerol, 20% 2-mercaptoethanol, 0.008% bromphenol blue, 0.25 M Tris HCl, all from Sigma-Aldrich) and incubated for 3 min at 95°C. Samples were separated by 10% SDS-PAGE and then transferred to a PVDF membrane (Amersham Pharmacia Biotech, Piscataway, NJ, USA). After 1 h of blocking in 1% BSA-TBST at room temperature, the membranes were incubated at 4°C overnight with specific primary antibodies for phospho-erk1/2 (Cell Signaling, Danver, MA, USA; 1:1,000), erk1/2 (Cell Signaling; 1:1,000), phospho-akt (Cell Signaling; 1:1,000), akt (Cell Signaling; 1:2,000), cPARP (Cell Signaling; 1:1,000), HIF-1α (Novus Biologicals, Littleton, CO, USA; 1:3,500), Caspase-3 (Santa Cruz, Heidelberg, Germany; 1:2,000) and ß-actin (Santa Cruz; 1:2,000). After washing in TBST buffer, the membrane was incubated for 1 h with peroxidase-conjugated swine anti-rabbit (Dako, Hamburg, Germany; 1:20,000) or peroxidase-conjugated rabbit anti-goat (Santa Cruz; 1:10,000) immunoglobulin G, referring to the manufacturer's instructions. Signals from peroxidase-conjugated antibodies were detected using the ECL kit (ECL-Plus Western blotting Detection Reagents, Amersham Pharmacia Biotech). Membranes were exposed to X-ray films and intensities of the protein bands were analyzed using the ImageJ software (v1.410, NIH). Signal intensities of P-erk1/2 and P-akt were relativized to the signals intensities of the total erk1/2 and total akt bands, signal intensities of Caspase-3 were relativized to the signal intensities of actin and signal intensities of the band for cleaved PARP (89 kDa) were relativized to the signal intensities of total PARP consisting of the bands of cleaved PARP (89 kDa) and uncleaved PARP (116 kDa).

### Quantification of Caspase-3/7 activity

Activities of the effector Caspases-3 and 7 were evaluated in cultures of IMR-32 cells using rhodamine based fluorometric assays (Apo-One homogeneous Caspase-3/7 assay, Promega Corporation, Madison, WI, USA). Treatment of the cells and evaluation of Caspase-3/7 activity were done on the basis of the manufacturer's protocol using a fluorescence ELISA reader (Genios FL; Tecan, Crailsheim, Austria) in combination with the Magellan software v1.1.

### Human apoptosis proteome profiler arrays

Proteome profiling was performed using commercially available human apoptosis proteome profiler arrays (R&D Systems, Minneapolis, USA) as per the manufacturer's protocol provided with the assay kit. Equal amounts (50 μg) of protein from each independently performed experiment (*N* = 5) were pooled and applied to the respective array membrane. Expression levels of 35 apoptosis-related proteins (effector and signaling molecules) were evaluated by densitometric analyses of the arrays using the ImageJ 1.41o software (ImageJ, NIH, USA). Proteins with expression levels of ≤10% of the intensity of the reference spots on the respective membrane were classified as unregulated. Regulation by 2-IB was only assumed if the protein of interest showed ≥25% up or down regulation.

### Statistical analysis

Statistical analyses were performed using GraphPad Prism 5 (GraphPad Software, San Diego, USA). All experiments were independently performed 3–5 times using at least duplicate samples. Data are presented as mean values with standard error of the mean (SEM). Statistical comparisons were performed using one-sample *t*-tests, one-way and two-way ANOVA with Bonferroni post-tests. Differences were considered to be statistically significant if P was less than 0.05. For parametric tests analyses, data were transformed (arcsin of square root of x) to obtain normality.

## Results

### Time kinetics of hypoxia-induced cell damage under hypothermic conditions

**Figure 2:**
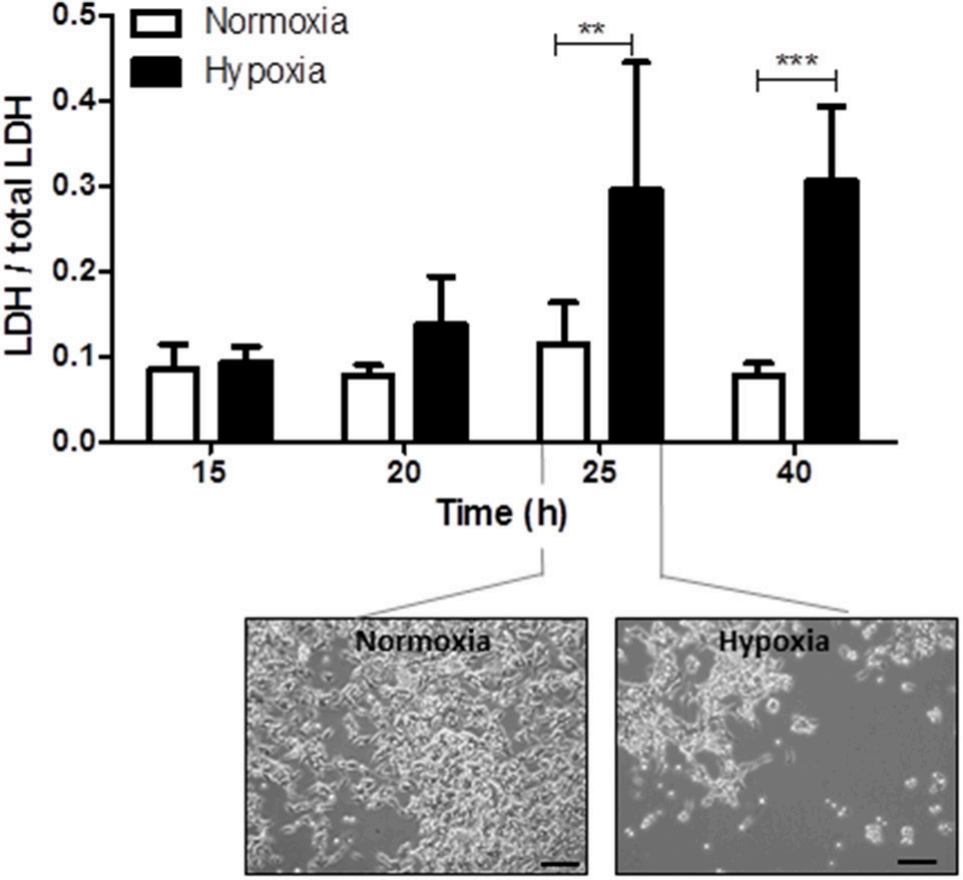
Time kinetics of hypoxia-induced cell damage under hypothermic conditions. Columns display the mean; bars denote SEM. Scale bars represent 100 μm. Horizontal lines above the columns assign groups with statistically significant differences (two-way ANOVA). ^**^P < 0.01; ^***^, *P* < 0.001; *N* = 3.

Reperfusion injury takes place several hours after the primary hypoxic insult. The optimal duration of reperfusion injury under hypothermic conditions was therefore determined by investigating cell damage, assessed by the release of LDH from cells into the culture medium, at 15, 20, 25, and 40 h after the end of hypoxia and normoxia, respectively. Significant cell damage was detected 25 and 40 h after the hypoxic insult (15 h after insult: hypoxia: 0.09 ± 0.02 and normoxia: 0.08 ± 0.03, *P* > 0.05; 20 h after insult: hypoxia: 0.14 ± 0.06 and normoxia: 0.08 ± 0.01, *P* > 0.05; 25 h after insult: hypoxia: 0.29 ± 0.14 and normoxia: 0.11 ± 0.04, *P* < 0.01; 40 h after insult: hypoxia: 0.31 ± 0.09 and normoxia: 0.08 ± 0.01, *P* < 0.001; Figure 2). Based on these findings, the shortest reperfusion time leading to significant cell damage (25 h) was chosen for all further experiments.

### Effects of 2-IB on hypoxia-induced cell damage, morphology and metabolic activity

**Figure 3:**
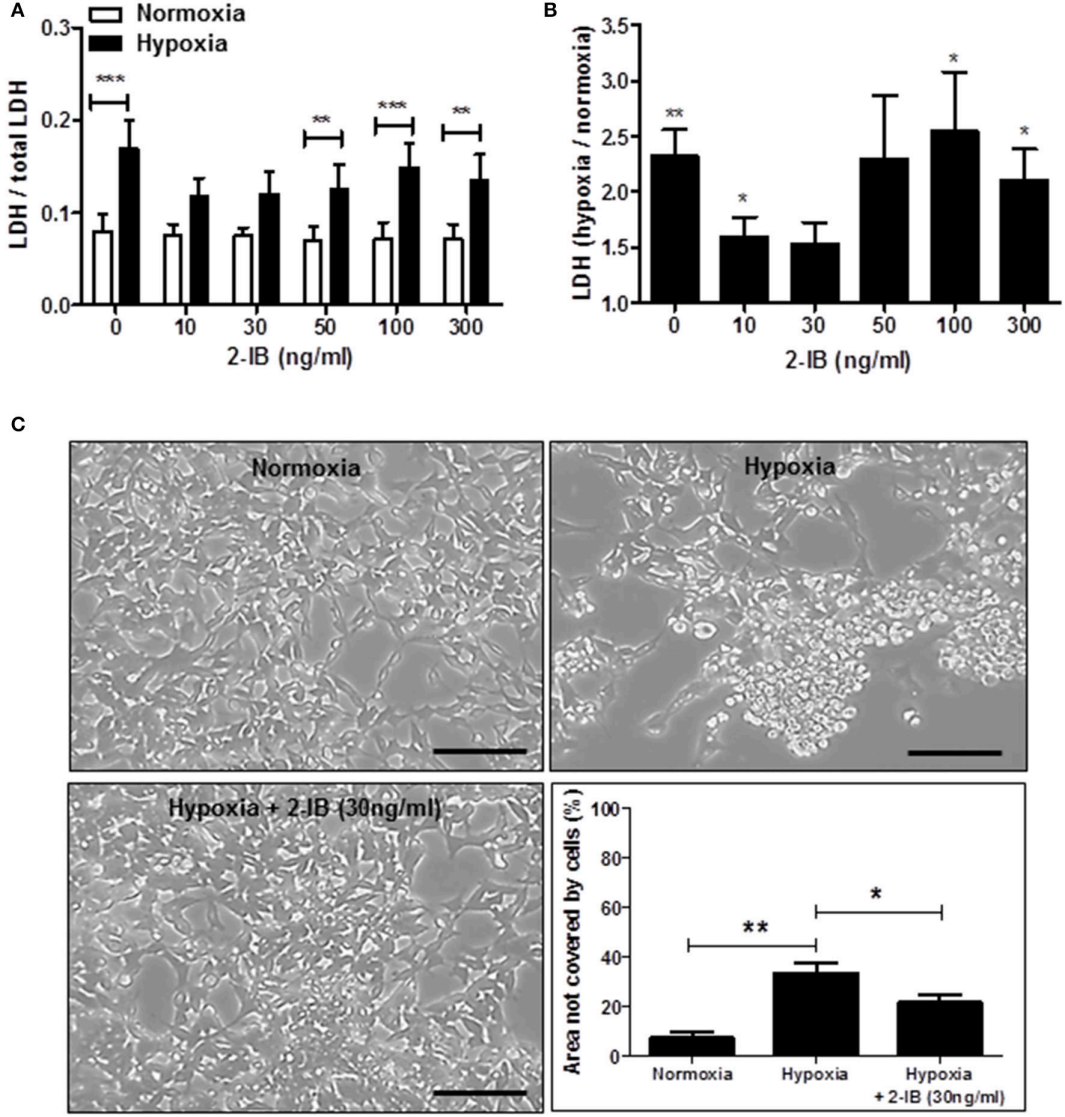
Effects of 2-IB on hypoxia-induced cell damage under hypothermic conditions. **(A)** LDH release under normoxic and hypoxic conditions. **(B)** Hypoxia-induced increase in LDH relative to the respective normoxia control (= 1). **(C)** Morphology of IMR-32 and morphometric quantification of the culture dish area not covered by cells. Columns display the mean; bars denote SEM. Scale bars represent 100 μm. Horizontal lines above the columns assign groups with statistically significant differences (two-way ANOVA in A, one-way ANOVA in C). Asterisks above the columns denote statistically significant differences compared to normoxia (one-sample *t*-test in **B**). ^*^P < 0.05; ^**^P < 0.01; ^***^P < 0.001; *N* = 5.

Application of low concentrations of 2-IB (10 ng/ml and 30 ng/ml) superimposed on hypothermia abrogated the hypoxia-induced LDH increase, resulting in LDH levels that were not different from the normoxia control. Interestingly, higher concentrations of 2-IB (50, 100, and 300 ng/ml) were not protective anymore, as LDH release was significantly increased by hypoxia compared to normoxia (Figure 3A): Without 2-IB, hypoxia: 0.17 ± 0.03 vs. without 2-IB, normoxia: 0.08 ± 0.02, *P* < 0.001; 10 ng/ml 2-IB, hypoxia: 0.12 ± 0.02 vs. 10 ng/ml 2-IB, normoxia: 0.07 ± 0.01, *P* > 0.05; 30 ng/ml 2-IB, hypoxia: 0.12 ± 0.02 vs. 30 ng/ml 2-IB, normoxia: 0.07 ± 0.01, *P* > 0.05; 50 ng/ml 2-IB, hypoxia: 0.13 ± 0.02 vs. 50 ng/ml 2-IB, normoxia: 0.07 ± 0.01, *P* < 0.01; 100 ng/ml 2-IB, hypoxia: 0.15 ± 0.03 vs. 100 ng/ml 2-IB, normoxia: 0.07 ± 0.02, *P* < 0.001; 300 ng/ml 2-IB, hypoxia: 0.13 ± 0.03 vs. 300 ng/ml 2-IB, normoxia: 0.07 ± 0.02, *P* < 0.01. Figure 3B displays the increase in LDH release relative to the respective normoxia control.

Post-hypoxic application of 2-IB (30 ng/ml) superimposed on hypothermia also attenuated the hypoxia-induced morphological signs of cell damage (e.g., cell rounding, retraction of cellular extensions, detachment from the growth surface; Figure 3C). Hypoxia significantly increased the area of the culture dish that was not covered by cells, even under hypothermic conditions (hypoxia: 33.40 ± 4.14% vs. normoxia: 7.23 ± 2.49%, *P* < 0.01), while application of 2-IB in combination with hypothermia attenuated the hypoxia-mediated increase of the area that was not covered by cells (hypoxia: 33.40% ± 4.14% vs. hypoxia + 30 ng/ml 2-IB: 21.38 ± 3.19%, *P* < 0.05; Figure 3C).

Measurements of metabolic activity revealed that the hypoxia-induced decrease in metabolic activity of IMR-32 cells was not affected by 2-IB (Supplemental Figure 1).

### Effects of 2-IB on hypoxia-induced ROS production and hydrogen peroxide release

**Figure 4:**
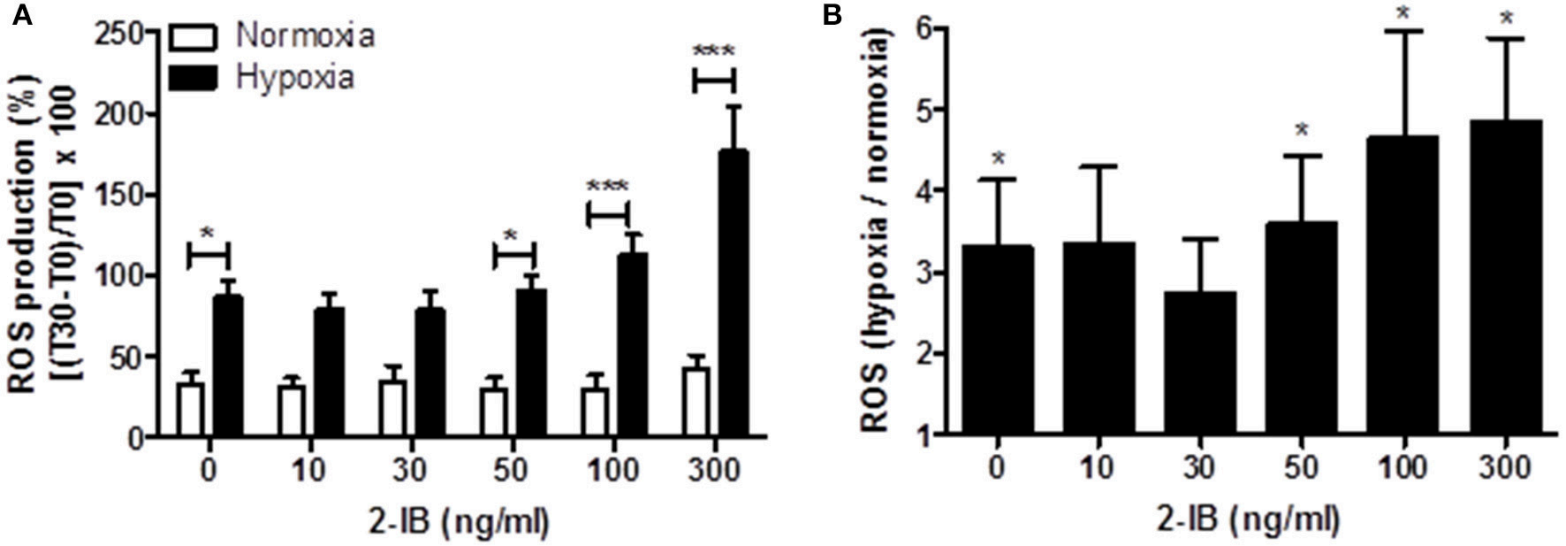
Effects of 2-IB on hypoxia-induced ROS production under hypothermic conditions. **(A)** ROS production under normoxic and hypoxic conditions. **(B)** Hypoxia-induced increase in ROS production relative to the respective normoxia control (= 1). Columns display the mean; bars denote SEM. Horizontal lines above the columns assign groups with statistically significant differences (two-way ANOVA in **A**). Asterisks above the columns denote statistically significant differences compared to normoxia (one-sample *t*-test in **B**) ^*^P < 0.05; ^***^P < 0.001; *N* = 5.

Application of low concentrations of 2-IB (10 and 30 ng/ml) superimposed on hypothermia did not effectively attenuate the hypoxia-induced ROS production, while higher concentrations (50, 100, and 300 ng/ml) of 2-IB even increased post-hypoxic ROS production (Figure 4A): Without 2-IB, hypoxia: 86.56 ± 10.45 vs. without 2-IB, normoxia: 32.86 ± 7.50, *P* < 0.05; 10 ng/ml 2-IB, hypoxia: 79.32 ± 9.94 vs. 10 ng/ml 2-IB, normoxia: 30.41 ± 6.35, *P* > 0.05; 30 ng/ml 2-IB, hypoxia: 78.41 ± 11.19 vs. 30 ng/ml 2-IB, normoxia: 34.19 ± 10.06, *P* > 0.05; 50 ng/ml 2-IB, hypoxia: 91.19 ± 9.43 vs. 50 ng/ml 2-IB, normoxia: 28.78 ± 8.33, *P* < 0.05; 100 ng/ml 2-IB, hypoxia: 112.50 ± 12.44 vs. 100 ng/ml 2-IB, normoxia: 29.39 ± 9.14, *P* < 0.001; 300 ng/ml 2-IB, hypoxia: 176.17 ± 27.82 vs. 300 ng/ml 2-IB, normoxia: 41.52 ± 9.18, *P* < 0.001). Figure 4B displays the increase in ROS production relative to the respective normoxia control.

Additional quantifications of culture medium concentrations of hydrogen peroxide revealed that although hydrogen peroxide levels were 9-fold increased by hypoxia under hypothermia (*P* < 0.001 vs. normoxia), post-hypoxic application of 2-IB did not influence the generation of hydrogen peroxide (Supplemental Figure 2).

### Effects of hypoxia and 2-IB on phosphorylation of Erk-1/2 and Akt

**Figure 5:**
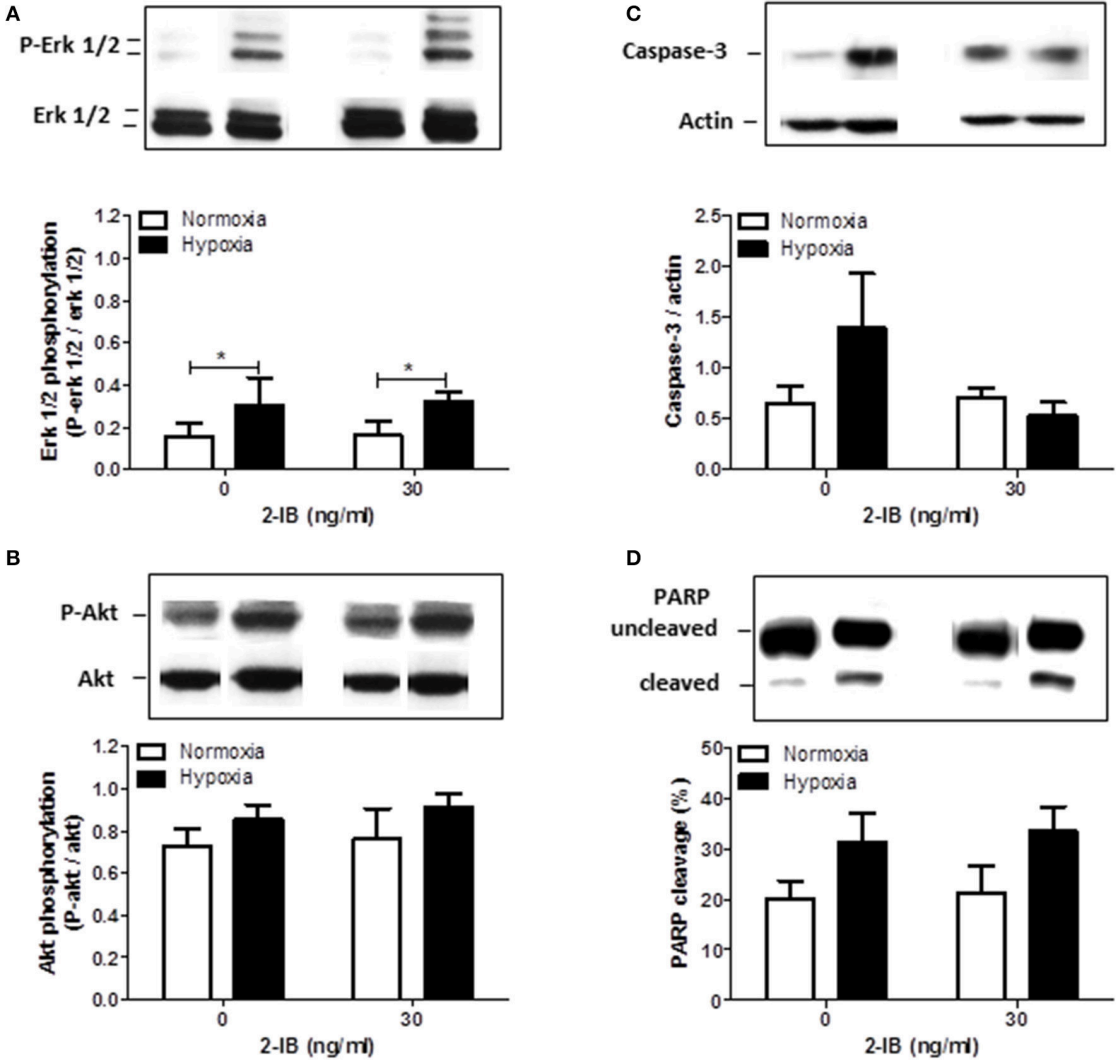
Effects of 2-IB on hypoxia-induced protein regulation under hypothermic conditions. **(A)** Erk1/2 phosphorylation. **(B)** Akt phosphorylation. **(C)** Caspase-3 expression. **(D)** PARP cleavage. Representative western blot bands are shown above the columns. Columns display the mean; bars denote SEM. Horizontal lines above the columns assign groups with statistically significant differences (two-way ANOVA) ^*^P < 0.05; *N* = 3.

Two hours after the hypoxic insult, increased phosphorylation of erk1/2, but not akt was detected under hypothermic conditions (P-erk1/2: hypoxia: 0.30 ± 0.07 vs. normoxia: 0.16 ± 0.03, *P* < 0.05). Application of 2-IB did not influence the phosphorylation of erk1/2 or akt (Figures 5A,B).

### Effects of hypoxia and 2-IB on Caspase-3 expression and cleavage of PARP

Twenty-five hours after the hypoxic insult, protein expression of Caspase-3 and cleavage of PARP were only slightly increased by hypoxia under hypothermic conditions. Addition of 2-IB did not significantly affect any of the investigated proteins (Figures 5C,D). Similar results were obtained by Caspase-3/7 activity assays (Apo-ONE assays; Supplemental Figure 3).

### Effects of hypoxia and 2-IB on apoptosis-related protein expression

As 25 h post-hypoxia might have been too late to detect apoptosis-related signaling and regulation of apoptotic pathways, total cell protein was obtained 4 h after hypoxia and analyzed for the expression levels of 35 apoptosis-related proteins using human proteome profiler arrays.

**Figure 6:**
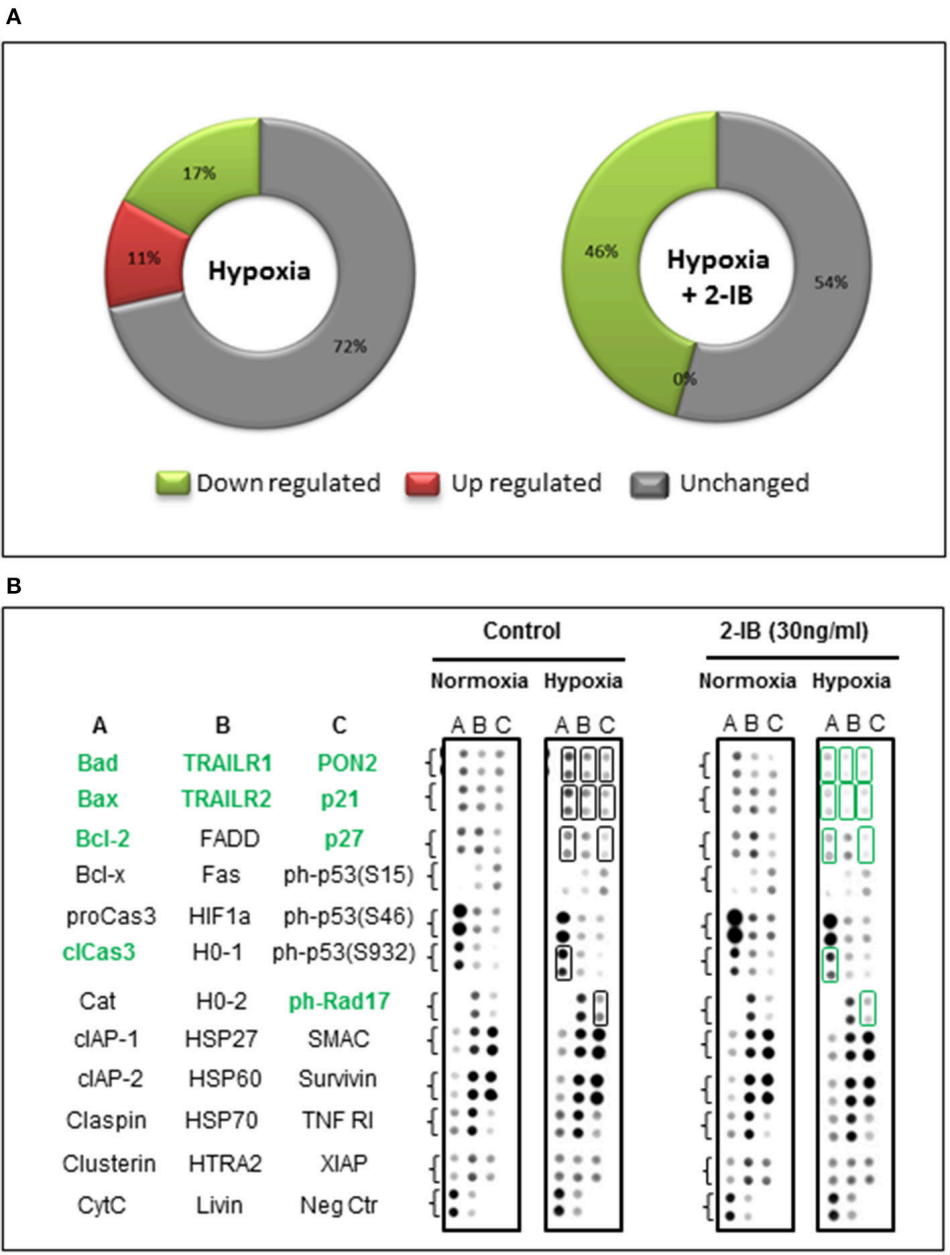
Effects of hypoxia and 2-IB on apoptosis-related protein expression under hypothermic conditions. **(A)** Effects of hypoxia on the expression of apoptosis-related proteins (compared to normoxia, left chart) and effects of post-hypoxic 2-IB application on the expression of apoptosis-related proteins (compared to normoxia + 2-IB, right chart). **(B)** Photomicrographs of the respective proteome profiling array. Proteins that are regulated ≥25% by 2-IB after hypoxia (hypoxia vs. hypoxia + 2-IB) are denoted in bold, green letters. Bad, Bcl-2 associated death promoter; Bax, Bcl-2 like protein 4; Bcl-2, B cell lymphoma-2; cleaved Caspase-3, cleaved Cysteine aspartic acid protease-3; TRAILR1, TRAIL receptor 1; TRAILR2, TRAIL receptor 2; PON2, Paraoxonase 2; p21, cyclin-dependent kinase inhibitor 1; p27, cyclin-dependent kinase inhibitor 1B; phospho Rad17, phosphorylated checkpoint clamp loader component. Samples from *N* = 5 independently performed experiments were pooled.

On the background of hypothermia, an up regulation of 4/35 (11%) apoptosis-related proteins was detected after hypoxia, while 6/35 (17%) were down regulated and 25/35 (72%) remained unchanged. Application of the protective concentration of 2-IB (30 ng/ml) resulted in 0/35 (0%) up regulated proteins. The numbers of down regulated apoptosis-related proteins increased to 16/35 (46%) while the expression levels of 19/35 (54%) proteins remained unchanged (Figure 6A). Compared to hypoxia, 10 apoptosis-related proteins were down regulated by more than 25% after post-hypoxic application of 2-IB (Bad, Bax, Bcl-2, cleaved Caspase-3, TRAILR1, TRAILR2, PON2, p21, p27, and phospho Rad17), while none of the investigated proteins was up regulated by more than 25% (Figure 6B). Raw data for the expression levels of each protein are shown in Supplemental Table 1.

## Discussion

Cooling is currently the state-of-the-art treatment for HIE. However, about 45% of infants continue to have an adverse outcome despite this treatment (Bel, 2016) and adjunct therapies on top of hypothermia are urgently needed to reduce the harmful effects of HIE. It has been described that hypothermia can influence drug pharmacokinetics and pharmacodynamics leading to reduced efficacy or even adverse drug events in the respective patients (Sunjic et al., 2015; Anderson et al., 2016). Therefore, the effectiveness of a specific drug to be intended for an adjunct therapy on top of hypothermia has to be carefully evaluated prior to its clinical application.

In the study presented, we have employed a human IMR-32 neuronal cell model in combination with our established enzyme-based hypoxia system (Huang et al., 2013a,b; Hummitzsch et al., 2014; Zitta et al., 2016) to mimic hypoxic-ischemic brain injury *in vitro* and evaluate whether 2-IB superimposed on hypothermia is able to protect human neuronal cells from hypoxia-induced cell damage.

Human IMR-32 cells used in our study originated from a metastatic site (Tumilowicz et al., 1970) and might not in all aspects resemble primary neuronal cells. However, they are commonly used neuronal cells that have been successfully employed in numerous *in vitro* experiments (Aldinucci et al., 2010; Huang et al., 2013a; Filatova et al., 2017). The *in vitro* hypoxia cell culture model is established in our laboratory since several years and reliably represents the main hypoxia-associated characteristics, such as (i) a decrease in pO_2_, (ii) a gradual reduction of glucose concentration and (iii) a decline of pH (Zitta et al., 2010a,b; Huang et al., 2013a,b).

In accordance with animal and clinical studies (Wagner et al., 2002; O'Brien et al., 2006; Galvao et al., 2013; Takenouchi et al., 2015; Diaz et al., 2017), our *in vitro* results suggest that hypoxia-induced cell damage can be attenuated by hypothermia (33.5°C). Additionally, our study data show that hypoxia still induces severe damage of neuronal cells even under hypothermic conditions. While in our previous study 7 h of hypoxia already led to significantly increased damage of IMR-32 cells after 17 h (Zitta et al., 2016), in our actual study using hypothermic conditions, significant cell damage was only observed 25 h post-hypoxia. Therefore, negative effects of hypoxia seem to be delayed by hypothermia but reappear at a later phase after the hypoxic insult. This observation once more underlines the need for additional treatment strategies superimposed on hypothermia.

*In vivo*, therapeutic hypothermia results in a reduction of metabolic activity (Zhu et al., 2015) and inhibition of pro-inflammatory cascades (Kimura et al., 2002; Hassell et al., 2015). As a direct consequence of decreased body temperature, global protein synthesis is repressed, switching the cellular program from cell growth to cell preservation (Sahuquillo and Vilalta, 2007). Although cellular metabolism is reduced by hypothermia, cells are still metabolically active to some extent (Erecinska et al., 2003), which may explain our *in vitro* observation that hypoxia-induced cell damage is only delayed but not prevented by hypothermia. This hypothesis is also supported by our preliminary findings, that under normothermic conditions, the amount of HIF1α, which represents a key molecule in hypoxia-induced cellular response, was increased as early as 2 h after hypoxia, while using the same setting, HIF1α levels were not different from baseline when hypothermia was established post-hypoxia (Supplemental Figure 4).

If superimposed on hypothermia, post-hypoxic application of different concentrations of 2-IB were able to reduce the hypoxia-induced cell damage 25 h post-hypoxia and our results suggest 30 ng/ml as optimal neuroprotective dose of 2-IB *in vitro*. In our previous cell culture study, hypoxia-mediated damage of neuronal cells was attenuated by 2-IB in the range between 30 and 50 ng/ml under conditions of normothermia (Zitta et al., 2016). In the actual study, which was performed on the background of hypothermia, neuroprotection by 2-IB was achieved at slightly lower concentrations (10–30 ng/ml). Interestingly, in both studies protective effects of 2-IB were only detected with low concentrations of 2-IB while the positive effects of 2-IB were abrogated at higher concentrations.

*In vivo*, using a piglet model, repeated 2-IB administrations of 6 × 1.0 mg/kg/dose *i.v*. resulted in neuroprotection with cerebrospinal fluid 2-IB concentrations of 24 ng/ml^14^. The close correlation of the neuroprotective 2-IB concentrations in animals and our human cell culture experiments supports the idea that similar concentrations of 2-IB (~30 ng/ml) could also reduce the detrimental effects of perinatal asphyxia in humans. Since impaired clearance is an important hindrance during hypothermia, it might however be necessary to change the dose regimen in clinical studies during hypothermia. Currently, only one small clinical trial (*N* = 12 patients) is investigating 2-IB as treatment option for newborn children suffering from perinatal asphyxia in addition to standard treatment with cooling (www.trialregister.nl: NTR5221). However, this study was designed to evaluate short term safety and tolerability of 2-IB and not protective effects of 2-IB, so that additional human trials are urgently needed.

Several studies suggested that 2-IB bears antioxidant potential (Peeters-Scholte et al., 2002c; Fan and van Bel, 2010; Tataranno et al., 2015). Our data showed that even under hypothermic conditions, production of ROS and hydrogen peroxide release were significantly increased by hypoxia. 2-IB did not effectively reduce the hypoxia-induced ROS production or hydrogen peroxide release under hypothermia. However, in our previous study, employing 2-IB under normothermic conditions, hypoxia-induced ROS production was to some extent reduced by 2-IB. Nevertheless, 50 ng/ml of 2-IB were necessary to obtain a slight reduction in ROS (Zitta et al., 2016).

The finding that low concentrations of 2-IB reduce hypoxia-induced cell damage, while these effects are abrogated by higher 2-IB concentrations is somewhat counter-intuitive. However, “U-shaped” dose-response curves of 2-IB are not only described in the actual study performed under hypothermia, but were also present when 2-IB was applied under normothermic conditions (e.g., “U-shaped” dose-response curve for LDH and inverted “U-shaped” dose-response curve for MTS measurements Zitta et al., 2016). Newer literature reveals growing evidence that “U-shaped” dose-response (hormesis) is commonly found in various biological systems and has been described for numerous different substances as well as different endpoints. Interestingly, essentially all pharmacologically based receptor systems display biphasic responses. Mechanistically, hormetic effects probably represent overcompensation in response to disruptions in homeostasis that are mediated by agonist concentration gradients with different affinities for stimulatory and inhibitory regulatory pathways (Calabrese and Baldwin, 2003; Calabrese, 2014; Zimmermann et al., 2014; Calabrese and Mattson, 2017).

Taken together, although ROS production and hydrogen peroxide release were significantly increased by hypoxia, no positive effects of 2-IB were detected. Therefore, we believe that an attenuation of ROS and hydrogen peroxide production and release are probably not the main mechanisms of 2-IB action.

Based on the above mentioned findings that 2-IB protects human neuronal cells from hypoxia-induced cell damage even if superimposed on hypothermia, we decided to investigate protein expression, cleavage and phosphorylation of several molecules involved in cell survival (erk1/2, akt) (Aksamitiene et al., 2012) and apoptosis (Caspase-3, PARP) (Dawson and Dawson, 2004). Phosphorylation of erk1/2 was significantly increased by hypoxia under hypothermic conditions, while phosphorylation of akt, protein expression of Caspase-3 and cleavage of PARP were also somewhat increased by hypoxia without reaching statistical significance. Addition of 2-IB at the cell protective concentration of 30 ng/ml did not affect any of the above mentioned molecules. Analysis of protein phosphorylation was performed 2 h post-hypoxia, while proteins involved in cell death and apoptosis (LDH, Caspase-3 and PARP) were analyzed 25 h after the hypoxic insult. We cannot exclude the possibility, that optimal time points for Caspase-3 expression/activity and PARP cleavage are earlier after hypoxia and effects of hypoxia, hypothermia and 2-IB on these apoptotic factors were overlooked in this setting.

To account for this limitation, total cell protein was obtained at an earlier time point (4 h post-hypoxia) and protein expression of 35 factors involved in apoptosis was evaluated using proteome profiler arrays. Our results show, that even under hypothermic conditions, hypoxia is able to change the expression levels of several apoptosis-related proteins. Post-hypoxic application of 2-IB resulted in a down regulation of 10 apoptosis-related proteins (Bad, Bax, Bcl-2, cleaved Caspase-3, TRAILR1, TRAILR2, PON2, p21, p27, and phospho Rad17), suggesting that the protective effects of 2-IB might be mediated at least partially by an early regulation of apoptotic pathways.

In summary, 2-IB superimposed on hypothermia reduces hypoxia-induced neuronal cell damage *in vitro*. Compared to the normothermic situation, slightly lower concentrations of 2-IB are sufficient to convey neuroprotection. Mechanisms of 2-IB involve an early regulation of several apoptotic proteins. We propose that hypothermia in combination with 2-IB might represent a potential treatment strategy for reducing hypoxia-induced neuronal cell damage and should be considered in future animal and clinical studies.

## Author contributions

Conception: KZ, CP-S, and MA. Data acquisition: KZ, LS, and KP. Analyses and interpretation: KZ, LS, KP, and MA. Drafting the manuscript for important intellectual content: KZ, CP-S, MG, LH, MS, and MA.

### Conflict of interest statement

CP-S is consultant for and shareholder of Neurophyxia BV's-Hertogenbosch, The Netherlands. The other authors declare that the research was conducted in the absence of any commercial or financial relationships that could be construed as a potential conflict of interest.
