# Supplementary figures and images for: 2-Iminobiotin Superimposed on Hypothermia Protects Human Neuronal Cells from Hypoxia-Induced Cell Damage: An in Vitro Study
Source: Front Pharmacol. 2018 Jan 11;8:971. doi: 10.3389/fphar.2017.00971 (PMC5768900; doi:10.3389/fphar.2017.00971)

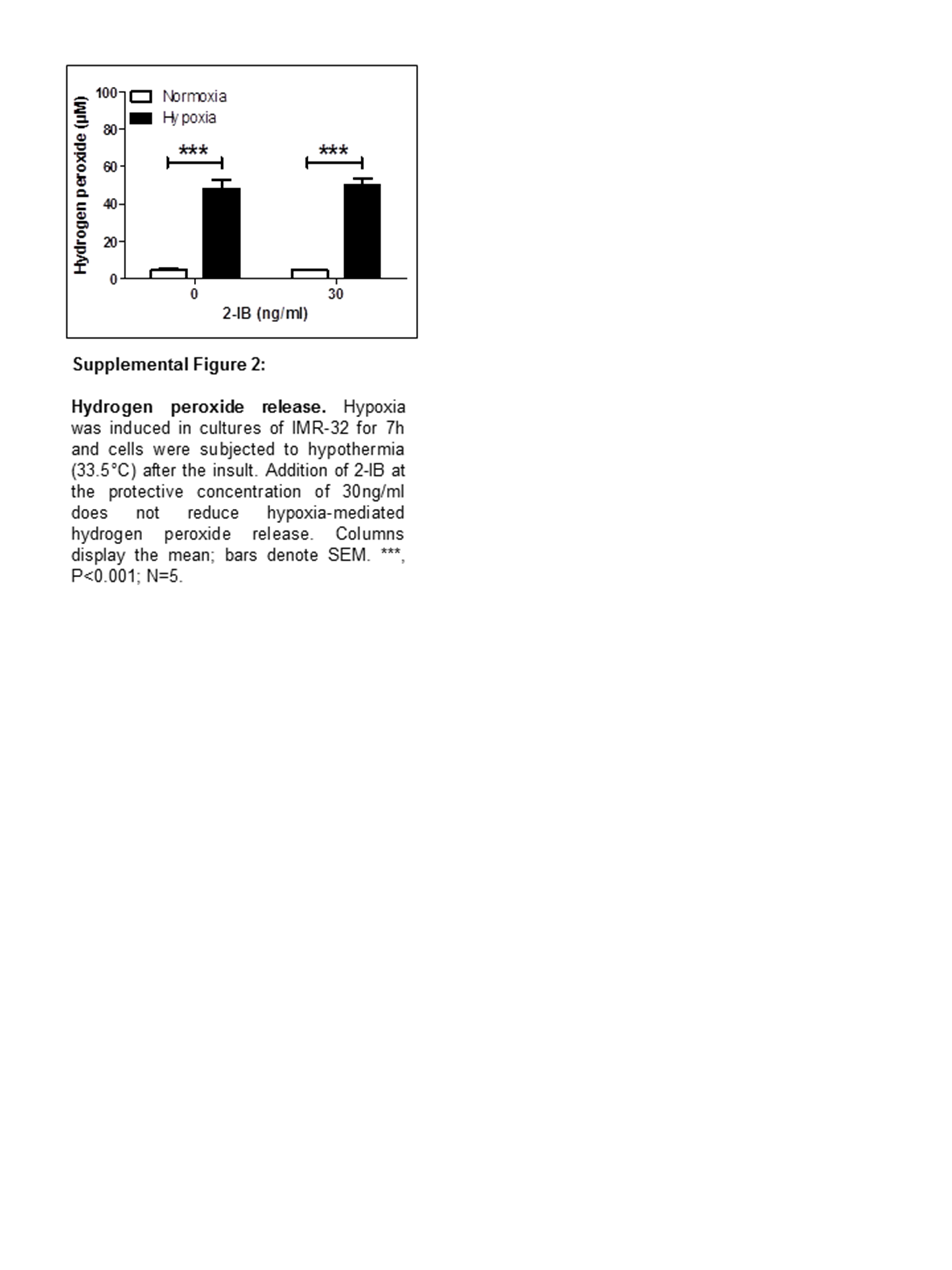

Supplement: Supplementary file 2 [file Image2.tif]

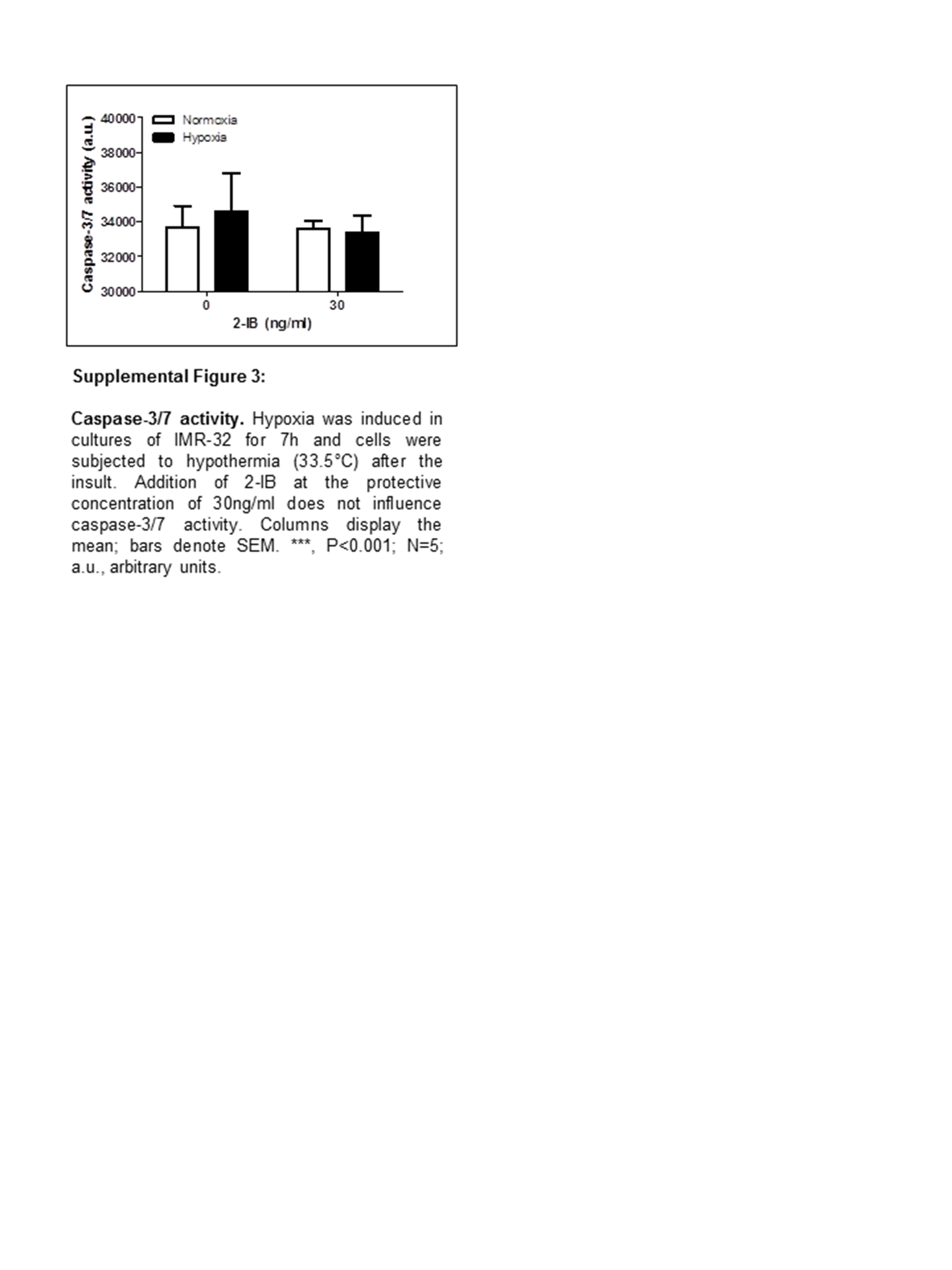

Supplement: Supplementary file 3 [file Image3.tif]

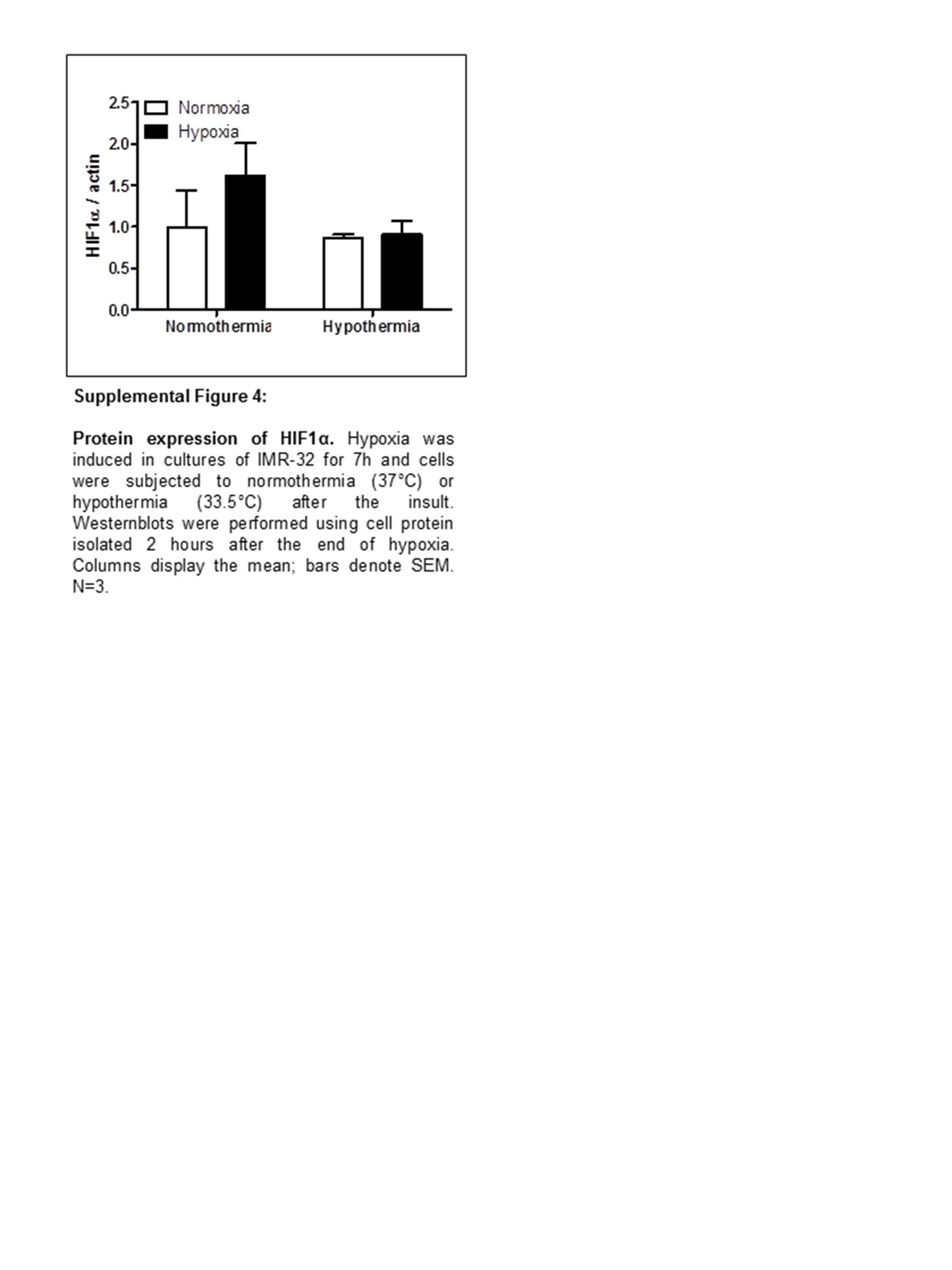

Supplement: Supplementary file 4 [file Image4.tif]
