# Supplementary material for: 2-Iminobiotin Superimposed on Hypothermia Protects Human Neuronal Cells from Hypoxia-Induced Cell Damage: An in Vitro Study
Source: Front Pharmacol. 2018 Jan 11;8:971. doi: 10.3389/fphar.2017.00971 (PMC5768900; doi:10.3389/fphar.2017.00971)
Supplement: Supplementary file 5 [file Table1.pdf]

	Control		2-IB (30ng/ml)	
	Normoxia	Hypoxia	Normoxia	Hypoxia
**Bad**	88	**91**	94	**38**
**Bax**	96	**96**	85	**40**
**Bcl-2**	90	**71**	79	**52**
Bcl-x*	22	12	9	18
Pro-Caspase-3	230	198	247	217
**Cleaved Caspase-3**	136	**124**	128	**92**
Catalase*	5	0	1	0
cIAP-1	46	60	61	55
cIAP-2	45	53	44	52
Claspin	73	78	64	64
Clusterin	56	63	64	59
Cytochrome C	130	115	129	113
**TRAIL R1 / DR4**	61	**59**	55	**22**
**TRAIL R2 / DR5**	81	**69**	90	**21**
FAAD	97	64	103	63
Fas / TNFRSF6 / CD95	49	35	46	30
HIF1α	78	54	96	62
H0-1 / HM0X1 / HSP32	53	51	69	48
H0-2 / HM0X2	89	115	111	97
HSP27	121	150	145	128
HSP60	166	151	178	147
HSP70	132	129	141	121
HTRA2 / 0MI	93	60	104	64
Livin	48	44	51	48
**PON2**	79	**50**	48	**28**
**p21 / CIP1 / CDKN1A**	65	**49**	70	**31**
**p27 / Kip1**	55	**38**	50	**26**
Phospho-p53 (S15)	75	66	78	57
Phospho-p53 (S46)	56	47	83	56
Phospho-p53 (S392)	28	27	40	32
**Phospho-Rad17 (S635)**	42	**72**	48	**46**
SMAC / Diablo	172	200	198	178
Survivin	194	193	201	156
TNF RI / TNFRSF1A	42	56	42	47
XIAP	66	62	84	65

Supplemental Table 1:

Analysis of proteome profiling of apoptosis-related proteins. Numbers in the table represent the mean densitometric intensities of duplicate sample spots. Proteins that are regulated ≥25% by 2-IB after hypoxia (hypoxia vs. hypoxia + 2-IB) are denoted in bold letters. *, proteins with expression levels ≤10% of the intensity of the reference spots. Note that several of the proteins are also influenced by 2-IB under normoxic conditions.
